# Spatial Differentiation Among Family Members of the Cooperatively Breeding Giant Babax Dynamically Adjusts to Temporal Fluctuations in Offspring Demand for Parental Care

**DOI:** 10.1002/ece3.73484

**Published:** 2026-04-09

**Authors:** Ning‐Ning Sun, Jian‐Chuan Li, Rang Li, Zhuo‐Feng Li, Rong‐Yu Xu, Miao Cheng, Shu‐Min Wang, Li‐Qing Fan, Bo Du

**Affiliations:** ^1^ School of Life Sciences Lanzhou University Lanzhou China; ^2^ Xizang Museum of Natural Science Lasa China; ^3^ Agricultural and Animal Husbandry University Linzhi China

**Keywords:** giant babax, GPS tracking, home range, movement pattern, spatial differentiation

## Abstract

In cooperatively breeding systems, spatial use and its associated resource acquirement pattern underlies parenting behavior of different group members. Therefore, quantifying spatial differentiation among group members is helpful to understand how parental care and cooperative breeding strategies evolve. In the giant babax ( *Babax waddelli* ), we used GPS tracking to quantify home range sizes and movement behaviors of dominant breeders and helpers, aiming to examine whether spatial differentiation occurred during different annual cycle phases. During the breeding phase when parental care is essential for offspring, helpers occupied larger home ranges and tended to move within higher‐altitude habitats than did dominant breeders. However, the temporal variation in movement patterns exhibited no significant difference between dominant breeders and helpers. During the postfledging phase when offspring require less parental care, helpers continued to occupy larger home ranges and move within higher‐altitude habitats; and the movement effort of dominant females was complemented by that of dominant males and helpers. During the wintering phase when offspring did not require parental care anymore, helpers were more likely to move in lower‐altitude habitats than dominant breeders, and no significant differences in the movement pattern were observed between dominant breeders and helpers. These findings provided evidence that spatial differentiation among family members of the giant babax dynamically adjusts in response to temporal fluctuations in offspring demand for parental care. Therefore, spatial differentiation may serve as the underlying mechanism for the diversification of parental care strategies within cooperatively breeding systems.

## Graphical abstract

This research provided evidence that division of labor indeed occurred when offspring were in need of parental care. Therefore, labor division may serve as the underlying mechanism for different parental care strategies and movement patterns among group members in the cooperatively breeding system of giant babaxes.

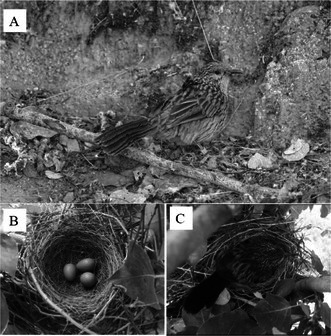

## 1 Introduction

In cooperatively breeding species, offspring are raised not only by the breeding pair (i.e., the dominant female and male) but also by nonbreeding adult group members known as helpers (Koenig and Dichinson 2004; Cockburn 2006). A substantial body of empirical evidence demonstrates consistent differentiation in parental care behaviors between dominant breeders and helpers, and between dominant females and males (Hamilton et al. 2005; Cram et al. 2015; Smith et al. 2024). The parental care differentiation represents a defining characteristic of cooperative breeding systems (Arnold et al. 2005; Beshers and Fewell 2001). Because an individual breeder's parental care strategy is closely linked to its resource use pattern (Gotwald 1974; Rueffler et al. 2012), comparative analyses of resource use among group members can yield critical insights into the evolutionary mechanisms shaping parental care strategies in cooperative breeders.

Traditional approaches for studying resource use rely on direct observation of adult parental care behaviors (Wiebe 2007; Ahern et al. 2011). However, such methods are often time‐consuming, labor‐intensive, and restricted to the breeding season. From a resource‐use perspective—particularly with respect to food acquisition—individual variation in resource use is frequently associated with consistent spatial preferences (Kurland and Beckerman 1985; Wahl 2002; Grinsted et al. 2013). When food resources are unevenly distributed, group members commonly exhibit spatial segregation across foraging areas, thereby reducing intragroup competition while enhancing individual foraging efficiency (Hegrenes 2001; Utsumi et al. 2009; Sassi et al. 2010; Hawlena et al. 2011). Given that spatial preferences can serve as a behavioral proxy for parental care, quantifying spatial use differentiation among group members—whether it reflects a cooperative strategy to reduce within‐group competition or results from dominant individuals monopolizing high‐quality habitats—provides a tractable alternative in advancing our understanding of how cooperative breeding strategies evolve.

A central concept regarding spatial preference is home range selection—the tendency of individuals to occupy a bounded area with all necessary limited resources supplied during a particular life‐history period (Burt 1943; Swihart and Slade 1985; Powell 2000). Accurately quantifying home range size, as well as movement behaviors within it, is essential for the investigation of spatial preferences (Duthé et al. 2023; Zhang et al. 2024; Brownlee et al. 2025). Early studies on home range rely on delineating boundaries of home range based on data of the spatial locations of trapped individuals (Hacker and Pearson 1946; Hayne 1949) or individual parenting behaviors (Guilford et al. 2008). However, such methods risk underestimating the true boundaries because animals can evade capture or witness (Allen 1943). The advent of radio telemetry has enabled automated recording of animal locations, but this technique can monitor tagged individuals within a confined range and requires recapture to retrieve data (Glenn et al. 2004; Dahle et al. 2006). GPS (global positioning system) tracking techniques provide a highly suitable approach for studying animal home ranges, because they facilitate long‐distance monitoring of animal movements and allow data to be collected continuously across time and space (Kotzerka et al. 2010; Ferrarini et al. 2018; Murgatroyd et al. 2023; Cheng et al. 2024; Dutta and Krishnamurthy 2025). However, due to constraints such as device weight and limited battery life, GPS loggers have not been widely adopted in studies of small‐bodied animals, particularly resident passerines that remain in restricted ranges year‐round.

**FIGURE 1:**
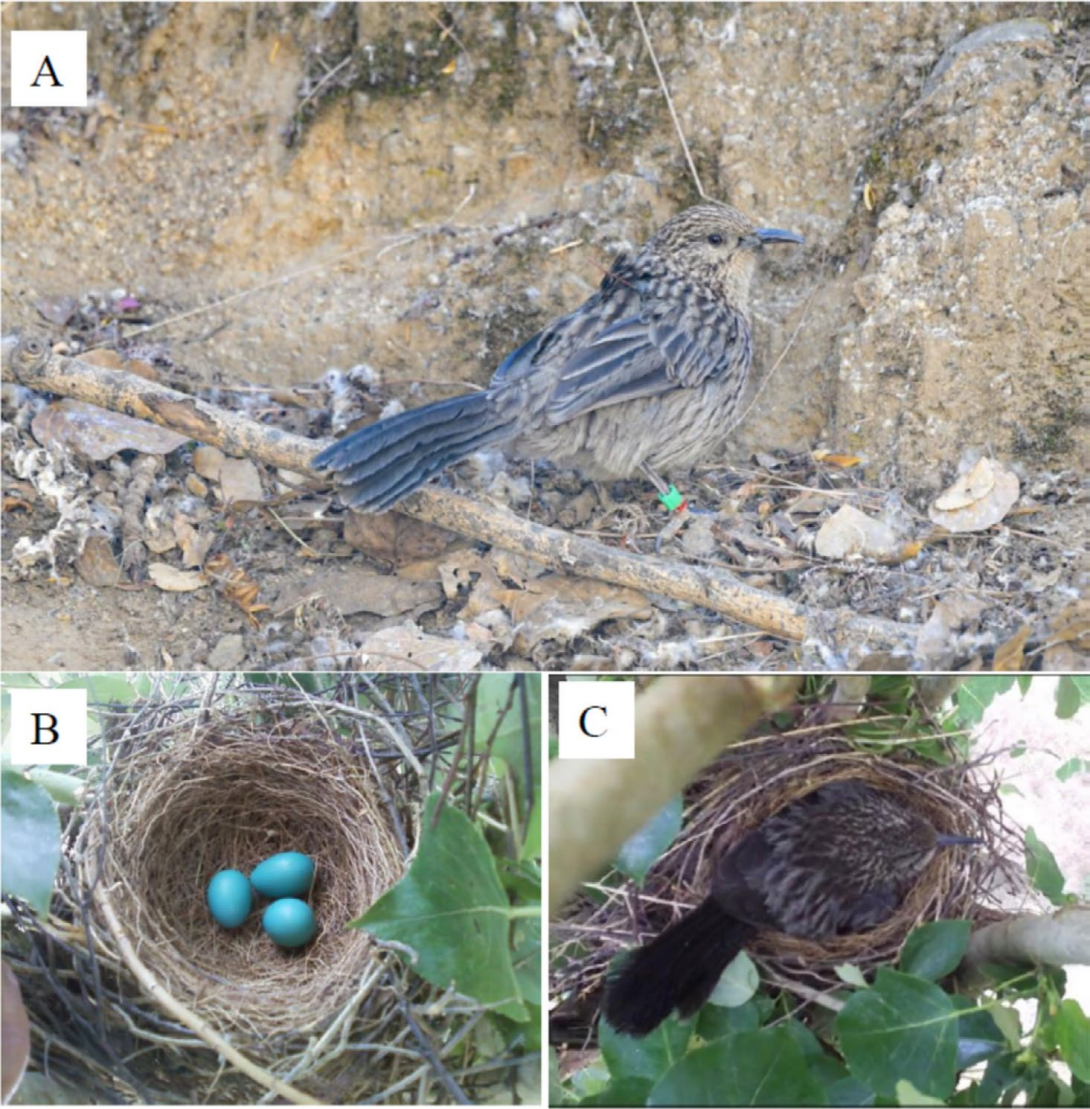
A tagged giant babax that foraged on the ground, which was taken by Zhuo‐Feng Li (A). (B) A nest with three eggs. (C) This individual is the dominant female as it brooded nestlings after food delivery.

The Giant babax ( *Babax waddelli* ) (Figure 1A), a member of the Timaliidae family (Aves, Passeriformes), is endemic to the Qinghai‐Xizang Plateau (Lu 2004). This species exhibits obligate cooperatively breeding, with all family units comprising helpers that are the offspring of the breeding pair, and helper numbers typically range from three to eight individuals (Du et al. 2012). Although clutches of six eggs have been occasionally documented, the typical clutch size is consistently three (Figure 1B), suggesting that some helpers delay dispersal for multiple breeding seasons (Liu et al. 2023). Within the extended family, helpers perform a range of tasks, including provisioning nestlings, defending the nest, and excluding both interspecific and intraspecific intruders from the territory (Du et al. 2012). During the nestling period, helpers contribute significantly to provisioning nestlings, with their total efforts significantly greater than the breeding pair (Fan et al. 2018). Although parental care diversification has been confirmed in the giant babax, it remains unclear what factors underlie this differentiation among group members.

In this study, we employed GPS tracking to assess seasonal variations in home range size and movement patterns among dominant female, dominant male, and helper giant babaxes across the breeding, postfledging, and wintering phases. Our objectives were to determine whether dominant breeders and helpers exhibit: (1) different provisioning rates and contributions to nestling provisioning during the nestling period, (2) divergent strategies in home range selection, (3) distinct approaches to spatial utilization with the home range, as reflected by vertical directions during movement, and (4) distinct movement patterns, characterized by locomotor speeds and activity levels.

## 2 Methods

### 2.1 Study Area and Population

This study was conducted in the Xiongse valley (29°44′ N, 91° 00′ E) in Qushui county, Xizang Autonomous Region, China, from November 2024 to September 2025. The altitude of this valley ranges from 3600 to 5000 m. The plateau's temperature semiarid climate in this region is characterized by hot in summer and cold in winter, with a large temperature difference between day and night and concentrated precipitation. Approximately 90% of the precipitation falls between May and September. Along with the ascendant height, landscapes change from hulless barley ( *Hordeum vulgare* ) farmland and its associated poplar (*Populus platyphylla*) to Lhasa barberry (*Berberis hemsleyana* Ahrendt) and Alpine willow (*Salix sclerophylla*) shrubberies, and to Tibetan Juniper (*Sabina tibetica*) forest and alpine scree vegetations. Giant babaxes prefer to construct their nests in the poplar at lower altitude and Lhasa barberry at higher altitude.

In the giant babax, the annual life‐history cycle can be delineated into three phases: breeding, offspring postfledging, and wintering. The breeding phase—defined as the interval from nest construction to offspring fledging—spanned from April 7 to May 11, 2025 across four monitored families. The offspring postfledging phase commenced once all offspring had fledged and concluded when juveniles achieved foraging independence, extending from May 12 to September 9 in this study. The wintering phase generally occurs from late October to late March of the subsequent year. For this study, data acquired between November 2 and December 22, 2025, were used to represent the wintering phase, as GPS loggers transmitted information exclusively during this interval.

### 2.2 GPS Tracking of Tagged Giant Babax

To precisely monitor the movement patterns of giant babaxes, 12 individuals from four families were captured using mist nets during ground‐foraging activities in mid‐November 2024. Prior to tagging, morphological measurements, including body mass (measured to 0.01 g), body length, and tarsus length (to 0.1 mm), were recorded from each adult individual. Tracking was subsequently conducted using backpack‐style GPS transmitters (model HQBG1202; Global Messenger Technology Co., Hunan Province, China). Each transmitter weighed 2.9 g, well below the 3% threshold of the birds' body mass, which ranged from 130 to 140 g (*n* = 20).

A dorsal attachment of the GPS transmitter was achieved using a flexible nylon cord threaded through the device's attachment loop. The cord was fastened to the scapular feathers under controlled tension, maintaining a gap of approximately 20 mm between the tracker and the body to prevent feather compression while ensuring both device retention and unimpeded flight performance. GPS units were programmed to acquire location data at hourly intervals, with data transmission initiated once five positional fixes had been stored.

All individuals were released immediately following the attachment of GPS transmitters. Their subsequent flight behavior was monitored using binoculars, with observational data confirming that tagged birds exhibited no signs of abnormal flight performance, such as lateral instability or diminished lift. Furthermore, giant babaxes were observed to spend the majority of their daily activity at foraging. Field re‐sightings confirmed that the species is resident and demonstrates strong site fidelity, as tagged individuals remained within the study area throughout the annual cycle. According to the design standards, these GPS trackers can be attached to individuals for at least 2 years. In fact, during the period of this study, no cases of drops occurred. However, due to the efficiency of charging, the data transmission process for most trackers was not continuous.

During the 2025 breeding phase, nests of the giant babax were identified through systematic surveys conducted within poplar and Lhasa barberry shrub habitats. Four active nests were monitored continuously over the breeding phase. GPS coordinates of all nests were documented to analyze their spatial distribution within the home range. During the egg period, incubation behavior of all group members was observed using binoculars. During the chick period, individuals visiting the nest to provision nestlings were captured using traps, following the protocol established by Ren et al. (2016). Each captured bird was fitted with a numbered aluminum leg band and two‐colored plastic rings to allow individual identification in subsequent behavioral observations.

If a tagged individual was recaptured during the breeding phase, its identity was determined based on brood match: female breeders have an obvious brood patch that covers the entire abdominal area, male breeders have a smaller brood patch covering approximately one‐third of the abdominal area, and helpers have no visible brood patch (Liu et al. 2023). If a tagged individual was not recaptured but was witnessed in behavioral observations using binoculars or in video recordings, its identity was verified according to its behaviors: female breeders typically carry out the full‐day incubation (Figure 1C) and brooding of nestlings, male breeders carry out incubation and brooding occasionally when female breeders were off the nest, and helpers never carry out incubation and brooding (Liu et al. 2023). Three categories of group members were recognized from these 12 tagged individuals: three female breeders (i.e., dominant females), three male breeders (i.e., dominant males), and four helpers. The remaining two individuals that were not captured or witnessed during the breeding phase were excluded in subsequent analysis.

To quantify parental care strategies of different group members, provisioning behaviors of adults during the breeding phase were recorded using ZX1 digital camcorders (Eastman Kodak Company), following the method of Liu et al. (2023). Data about adult provisioning behaviors were extracted from the videos by playing back on the computer. The identity of a nest visitor was first identified by its leg bands (if yes) or behaviors (if no leg bands), with the dominant female usually brooding the nestlings after food delivery, the dominant male often courtship feeding the brooding female, and helpers just delivering food to nestlings (Liu et al. 2023). Other data included: (1) the start and end time of a feeding event, (2) the number of nest visitors in a feeding event, and (3) the duration of a video recording. Based on these data, we calculated the provisioning rate of a tagged individual as the number of feeding event it carried out per hour. Moreover, the relative contribution of dominant female or male to nestling provisioning was calculated as the proportion of feeding events it carried out in a video recording. As there were multiple helpers in giant babax families, the contribution of helpers to nestling provisioning was calculated as the proportion of total feeding events all helpers carried out in a video recording. In this study, we obtained 181 h of video recordings collected from 14 giant babax nests (mean ± SEM, 17.935 ± 1.622 h, *n* = 14 nests).

### 2.3 Data Curation

The retrieved tracking data comprised timestamp (GMT + 8), longitude, latitude, altitude (m, ASL), position accuracy, instantaneous locomotor speed (km/h), and activity level. Position accuracy refers to the proximity between a satellite‐derived position and its true location on Earth. Activity level was derived from triaxial accelerometer readings: during each sampling cycle, the cumulative count incremented by 1 whenever acceleration exceeded 0.15 g in any axis (*x*, *y*, or *z*), aligning with avian movement models (Shepard et al. 2008; Halsey 2011; Halsey et al. 2015). The dates of each phase were converted into a continuous variable (named seasonal days), with the earliest day assigned as 1. Each day's time was standardized as a continuous variable (named diurnal time), starting from one at zero o'clock.

Home range sizes of these 10 tagged individuals in different seasons were calculated from 95% kernel density estimations (KDE) based on utilization distribution (Worton 1989), using the “rhrKDE” function in the R package “rhr” (Signer and Balkenhol 2015). Their core area sizes were also obtained from 50% KDE.

### 2.4 Statistical Analysis

Because the habitat of giant babaxes in our study area is located on a steep slope, their daily activity areas are not on a single plane. To examine whether dominant females, dominant males and helpers differed in their position altitude during daily activities, we fitted a generalized linear mixed model (GLMM) to account for no independence arising from repeated sampling of the same individuals across days. Position altitude was modeled as the normally distributed response variable with an identity link function. “Individual category” (dominant female, dominant male, helper) was included as a fixed effect variable, and individual identity (i.e., unique tag ID) was specified as a random effect to control for among‐individual variation.

To examine the variation of relative contributions of dominant male, dominant female, and helpers to nestling provisioning with nestling age, GLMMs were fitted to control for the repeated sampling of the same individual across different days. The relative contribution was first transformed using the square root of the arcsine before setting it as the dependent variable. It was normally distributed, and thus an identity link function was employed. Nestling age was set as the fixed effect variable, and the label of nests was set as the random effect variable.

To examine whether tagged individuals exhibited different movement patterns, GLMMs were fitted by setting the locomotor speed or activity level during each annual cycle phase as the response variable, with normal distribution and an identity link function. Fixed effect variables included the category of tagged individuals and its interplay with seasonal days and diurnal time. The identity label of tagged individuals was set as the random effect variable to control for the repeated sampling of the same individuals across different days.

Data used in the GLMM analyses could be accessed in Sun et al. (2026). GLMM analyses and associated linear regression were performed using the package *lme4* in R (version 4.4.3, R Core Team 2018). The visualization of the statistical results was accomplished by *ggplot2* (Wickham 2016). Descriptive results are presented as mean ± SEM, and overall significance was evaluated based on *p*_two‐tailed_ < 0.05, indicating rejection of the null hypothesis.

## 3 Results

By employing GPS tracking, we quantified the home range size, altitude during locomotion, movement speed, and activity level of giant babaxes during a specific life‐history phase. Comparisons of these metrics between dominant females/males and helpers revealed that members within the cooperatively breeding system of giant babaxes adopted distinct strategies in both home range selection and movement patterns.

### 3.1 Variation in Home Range Size and Altitude Over Seasons

**FIGURE 2:**
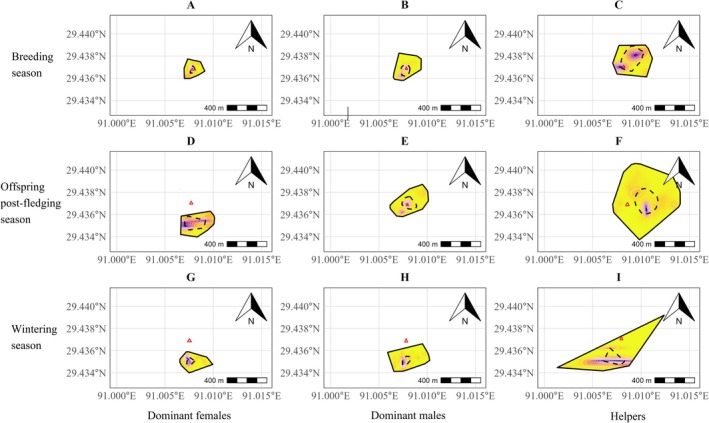
Quantifying and comparing home range sizes of tagged giant babaxes in breeding (dominant females, 2.59 × 10^5^ m^2^; dominant males, 6.95 × 10^5^ m^2^; and helpers, 16.80 × 10^5^ m^2^), offspring postfledging (dominant females, 3.46 × 10^5^ m^2^; dominant males, 10.19 × 10^5^ m^2^; and helpers, 20.22 × 10^5^ m^2^), and wintering season (dominant females, 5.02 × 10^5^ m^2^; dominant males, 6.19 × 10^5^ m^2^; and helpers, 14.25 × 10^5^ m^2^). The irregular circular rings within the home range represent contour lines, indicating that the higher the altitude, the farther the distance away from the nest site. Red triangles indicating the nest site of a family occupied during the breeding phase.

By quantifying and comparing the home ranges sizes of different group members (Figure 2), it appeared that dominant females occupied the smallest area while helpers occupied the largest one during the breeding (Figure 2A–C), postfledging (Figure 2D,E), and wintering phase (Figure 2G–I). All individuals had the smallest home range during the breeding phase while the largest one during the postfledging phase (Figure 2). During the breeding phase, the ranges of dominant females' and males' moving tended to be centered around the nest site, while the ranges of helpers' moving were farther away from the nest site (Figure 2A–C).

Regarding the altitude at which a tagged individual was positioned, it differed significantly between dominant breeders and helpers during the breeding phase but not during the postfledging or wintering phases (Table S1). Dominant females and males moved at significantly lower altitude than did helpers during the breeding phase, whereas no significant differences were observed during the postfledging and wintering phases (Table S1).

### 3.2 Provisioning and Movement Patterns During the Breeding Phase

**FIGURE 3:**
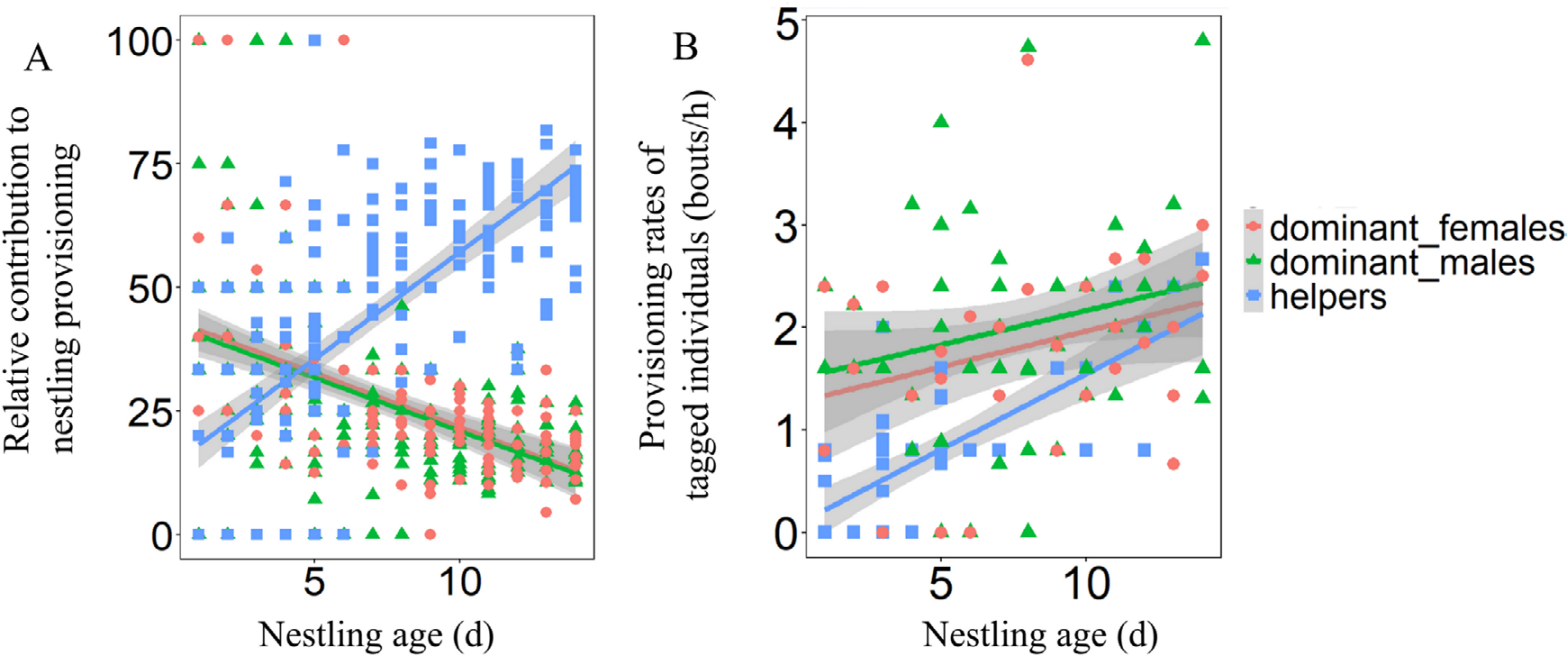
Temporal variations in relative contributions of different group members to provisioning nestlings (A) and their provisioning rates (B) with nestling age in the giant babax.

The relative contributions of all group members to nestling provisioning were significantly associated with nestling age (Table S2). As nestlings grew, dominant males and females reduced their contributions, while helpers significantly increased their contributions in the provisioning of nestlings (Figure 3A). Regarding the provisioning rates, tagged dominant males (*R* ^2^ = 0.058, *F*_1,52_ = 3.179, *p* = 0.080) and females (*R* ^2^ = 0.091, *F*_1,32_ = 3.198, *p* = 0.083) exhibited a tendency to increase the provisioning rates with nestling age, while tagged helpers significantly increased their provisioning rates with nestling age (*R* ^2^ = 0.530, *F*_1,44_ = 49.578, *p* < 0.001; Figure 3B).

**FIGURE 4:**
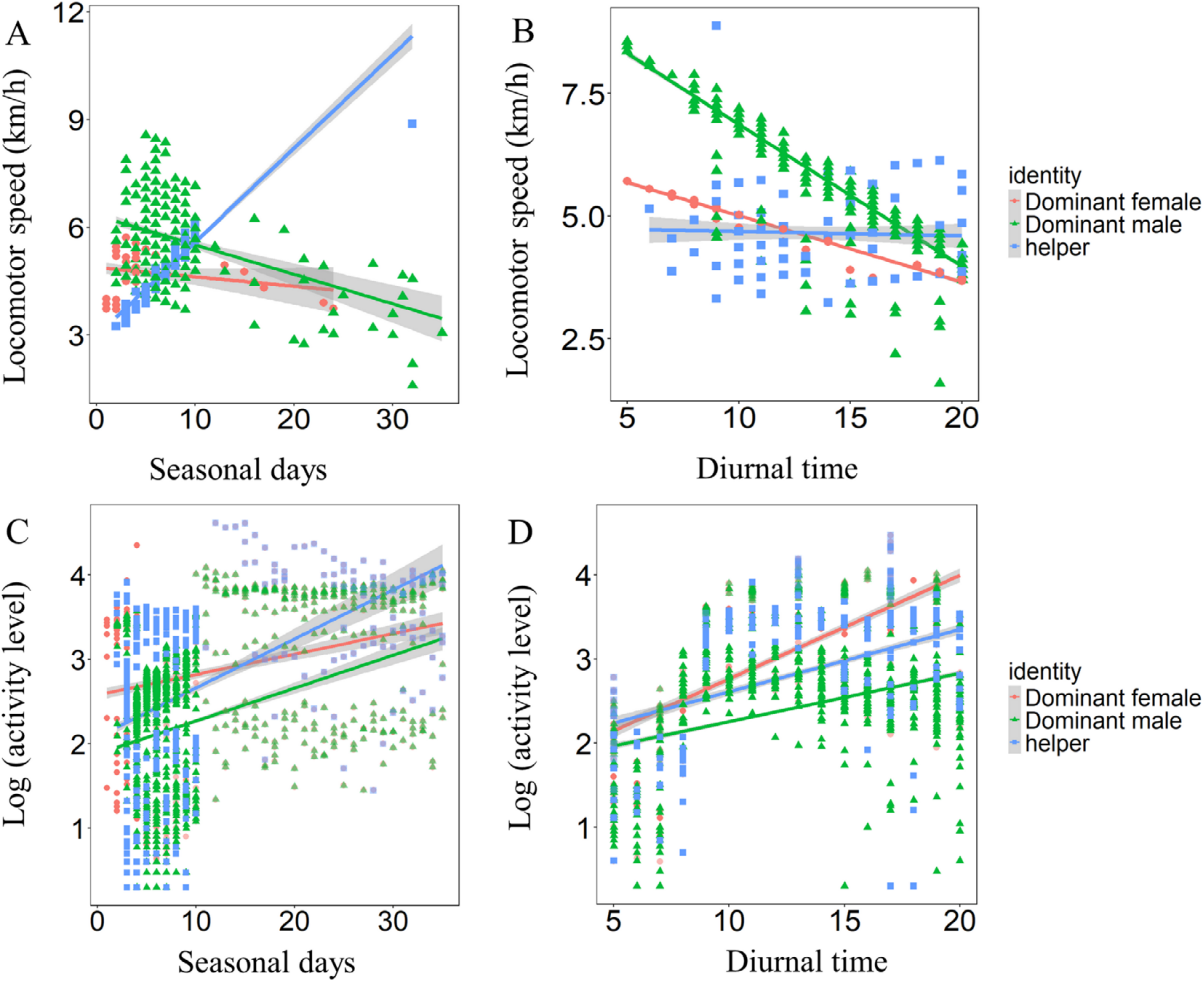
Variations in the movement patterns of tagged giant babaxes during the breeding phase.

During the breeding phase, the locomotor speeds of tagged giant babaxes exhibited no significant differences between dominant females/males and helpers (Table S3). Dominant females and males did not change their locomotor speeds with seasonal days while helpers tended to increase their locomotor speeds with seasonal days (Figure 4A and Table S3). Dominant females and helpers did not change their speed with diurnal time while dominant females decreased their locomotor speed significantly with diurnal time (Figure 4B and Table S3).

The activity level of tagged giant babaxes exhibited no significant differences between dominant females/males and helpers during the breeding phase (Table S4). All tagged individuals significantly increased their activity levels with seasonal days (Figure 4C) and diurnal time (Figure 4D).

### 3.3 Movement Patterns During the Postfledging Phase

**FIGURE 5:**
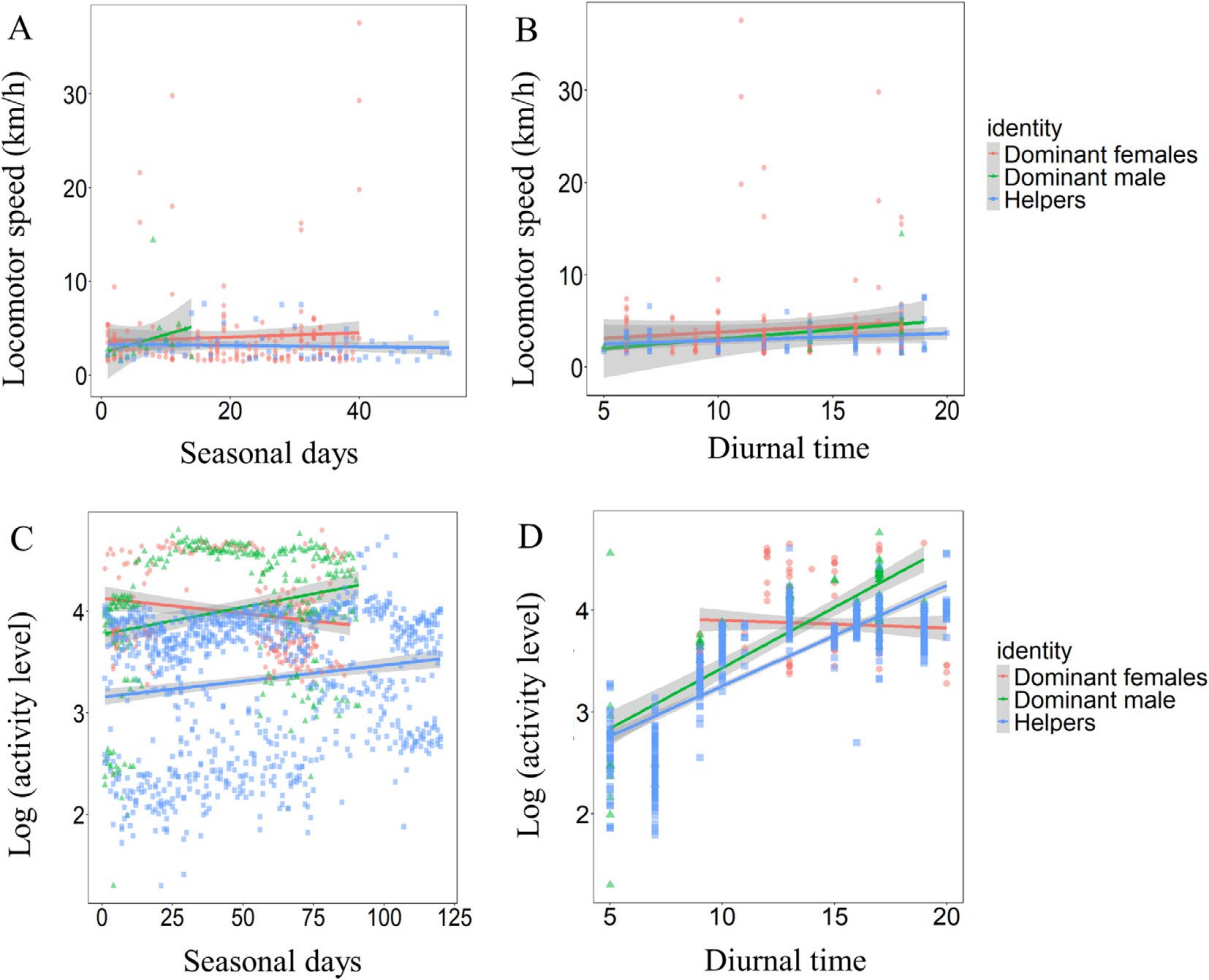
Variations in the movement patterns of tagged giant babaxes during the offspring postfledging phase.

During the offspring postfledging phase, the locomotor speeds of tagged giant babaxes exhibited no significant differences between dominant females/males and helpers (Table S5). All tagged individuals did not change their locomotor speeds significantly with seasonal days (Figure 5A). Dominant females exhibited a weak tendency to increase locomotor speed with diurnal time whereas dominant males and helpers exhibited no such tendency (Figure 5B and Table S5).

The activity levels of tagged dominant females and males were both significantly higher than that of tagged helpers (Table S6). Dominant females significantly decreased while dominant males and helpers increased their activity levels with seasonal days (Figure 5C and Table S6). Similarly, dominant females significantly decreased while dominant males and helpers increased their activity levels with diurnal time (Figure 5D).

### 3.4 Movement Patterns During the Wintering Phase

**FIGURE 6:**
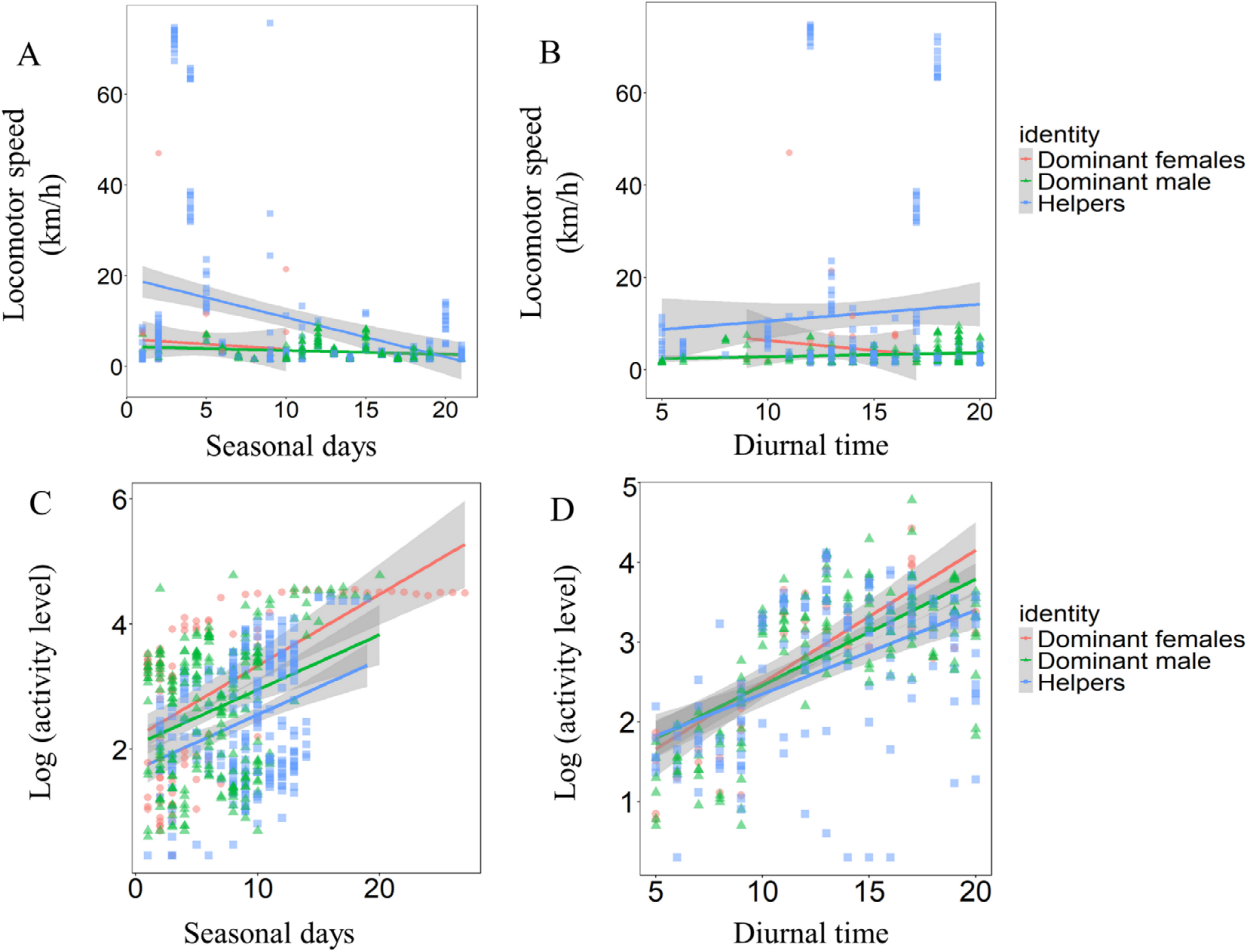
Variations in the movement patterns of tagged giant babaxes during the wintering phase.

During the wintering phase, the locomotor speeds of tagged giant babaxes exhibited no significant differences between dominant females/males and helpers (Table S7). Dominant females and females did not change locomotor speed significantly with seasonal days (Figure 6A) and diurnal time (Figure 6B). Helpers significantly decreased locomotor speed with seasonal days (Figure 6A) while increased locomotor speed with diurnal time (Figure 6B and Table S7).

The activity level of giant babaxes during the wintering phase exhibited no significant differences between dominant females/males and helpers (Table S8). All tagged individuals significantly increased their activity levels with seasonal days (Figure 6C and Table S8) and diurnal time (Figure 6D and Table S8).

## 4 Discussion

By utilizing GPS tracking to automatically monitor the movement behaviors of 10 tagged individuals, we provided evidence for home range differentiation between dominant breeders and helpers in the cooperative breeding system of giant babaxes. Movement patterns of all group members exhibited dynamic adjustment across distinct phases of the annual cycle, particularly aligning with temporal shifts in offspring demand for parental care.

### 4.1 Spatial Differentiation Optimizing the Efficiency of Nestling Provisioning During the Breeding Phase

In cooperative breeding systems, diverse parental care strategies have been widely reported among group members (Hatchwell 1999; Cockburn 2006). This diversity might have resulted from a negotiation regarding reproductive shares, with members obtaining greater reproductive shares providing more parental care for offspring (Kokko et al. 2002; Magrath et al. 2004; Robertson et al. 2018). Alternatively, diverse parental care strategies may be closely linked to spatial differentiation, where dominant breeders and helpers differentiate in their utilization of space and food types (Shimoji and Dobata 2022). In the giant babax, results about adult provisioning behaviors confirmed that dominant breeders and helpers adopted distinct parental care strategies during the breeding phase (Figure 3). As helpers occupied larger home ranges (Figure 2A–C) and they moved more frequently within higher‐altitude habitats than dominant breeders (Table S1), it suggests that dominant breeders and helpers employed distinct space‐use strategies. Consequently, spatial differentiation among group members may be an effective method for giant babaxes to optimize their cooperative strategy.

During the breeding phase when offspring are most dependent on parental care, spatial differentiation and divergent movement patterns between dominant breeders and helpers can enhance the efficiency of nestling provisioning. As dominant females mainly undertake the duty of incubating eggs and brooding nestlings, spending more time near the nest and occupying smaller home ranges can reduce travel distance and time to and from the nest. Dominant males, which take the greatest share of parental duties—including courtship feeding (Fan et al. 2018), must acquire greater quantities of food per unit time. A smaller home range can minimize transit time and increase foraging efficiency. By contrast, the consistent high number of helpers in the giant babax allows their parenting workload to be distributed across multiple individuals. By exploiting larger home ranges and higher‐altitude habitats, helpers can reduce spatial overlap with dominant breeders while increasing access to underutilized food resources near range boundaries. Therefore, these complementary spatial use strategies reflect an evolutionarily refined cooperative system optimized for reproductive success.

### 4.2 Effects of Offspring Varying Demands for Parental Care on Adult Movement Patterns Across Distinct Phases

Variations in individual conditions can result in behavioral diversification among group members (Lendvai et al. 2004, 2006; Krebs 2014). For example, in flock‐feeding house sparrows ( *Passer domesticus* ), hungry individuals exhibit a greater propensity for scrounging than satiated ones (Lendvai et al. 2004), and dominant individuals consistently adopt a scrounging strategy while subordinates do so only opportunistically (Lendvai et al. 2006). Under energetically challenging conditions, such as the high‐altitude environments inhabited by giant babaxes, considerations of energy conservation—in addition to the demands of parental care—may further shape movement patterns in this species.

During the breeding phase, despite the presence of helpers, dominant females and males generally maintained their movement efforts, with no significant reduction observed in response to helper assistance (Figure 4), with the exception for dominant males that exhibited a progressive decrease in locomotor speed over diurnal time (Figure 4B). This pattern may be explained by the persistent parental care demands of offspring, which likely prevented dominant breeders from fully relying on helpers to provision nestlings. As nestlings develop and their nutritional needs escalate, dominant breeders are driven to intensify their movement activity to meet the increasing demands (Figure 4C,D). Consequently, the interplay between offspring care requirements and the necessity of providing parental care collectively shapes adult movement patterns during the breeding phase.

During the offspring postfledging phase, as offspring progressively reduce their dependence on parental provisioning, dominant females exhibit a gradual decline in movement effort with advancing seasonal days and diurnal time (Figure 5C,D), thereby offsetting their substantial energetic investment during breeding. In contrast, both dominant males and helpers increase their movement activity to compensate for the reduced contribution of dominant females, ensuring that newly fledged juveniles receive a consistent level of food provisioned by all caregivers.

During the wintering phase, when adults are free from offspring provisioning, helpers exhibited a tendency to reduce locomotor speed over seasonal days (Figure 6A); and all tagged individuals exhibited the same trend of increasing movement activity with seasonal and diurnal time (Figure 6C,D). As food resources become progressively depleted during winter, the consistent movement pattern among group members suggests that all individuals must increase movement effort to secure sufficient food for overwinter survival.

Regarding the mechanisms of spatial preferences during home range selection between dominant breeders and helpers, the observed pattern likely reflects a negotiation based on the types of assistance provided by helpers. Given that helpers perform a substantial proportion of territory defense (Liu et al. 2023), they are required to patrol territorial boundaries frequently. As a result, by positioning themselves farther from the nest site, helpers occupy significantly larger home ranges than dominant breeders. Moreover, because helpers are generally the offspring of dominant breeders, we observed no aggression from dominant breeders toward helpers during their daily interactions. Therefore, spatial differentiation in this species appears not to be driven by aggressive displacement by dominant breeders through occupying higher‐quality habitats. The significance of applying GPS tracking in the giant babax.

In cooperatively breeding systems, the presence of helpers frequently induces dominant breeders to reduce their investment in parental care (Heinsohn 2004; Zöttl et al. 2013; Adams et al. 2015; Hirokazu et al. 2018; Mermoz et al. 2025). However, the challenges of monitoring individual behavior under natural conditions have limited our understanding of how dominant breeders and helpers coordinate spatially and behaviorally to optimize resource use. To overcome the challenges associated with behavioral monitoring, GPS tracking offers a powerful tool to continuously monitor and quantify the movement behaviors of multiple individuals. Therefore, GPS tracking has been increasingly employed in movement ecology studies of cooperatively breeding (Ferreira et al. 2024) or biparental breeding birds (López‐López et al. 2021; Ferreira et al. 2024; Becciu et al. 2025; Lok et al. 2025). By using GPS tracking to investigate home range and movement behaviors of the giant babax, our study also demonstrates its utility for monitoring fine‐scale daily activities in resident species. This approach thereby expands the potential applications of GPS tracking in future behavioral research on small‐bodied passerines.

### 4.3 Limitations of the Present Study

Obviously, the largest shortcoming of this study is attributed to the small sample size. Given the endangered status of the giant babax and the expensiveness of GPS loggers, the number of tagged individuals was restricted in this study. As the giant babax is sexually monomorphic in plumage and size, it was difficult to identify the sex of tagged individuals during winter. The random selection of 12 individuals acquired three dominant females and three dominant males from four families. Therefore, although helpers exhibited a tendency of moving within larger home ranges than did dominant breeders in all three seasons (Figure 2), this difference did not reach a significant level. The generality of this finding still requires further validation using a larger sample size.

Another limitation of this study is the low coverage in sampling helpers because there are at least five helpers in an extended family of the giant babax. The random selection of individuals when fixing GPS loggers resulted in tracking of at most two helpers within a family. Considering that different helpers may undertake different tasks (Fan et al. 2018), the home range selection and movement patterns revealed in this study might not accurately represent the behavioral characteristics of all helpers. Furthermore, the inability to determine the sex of helpers weakened our understanding of the labor division among multiple helpers within the same family. Likewise, the crucial approach to addressing this shortcoming is to increase the sample size.

## 5 Conclusion

By employing GPS tracking to monitor home range and movement behaviors of dominant breeders and helpers across distinct annual cycle phases, this study confirms the occurrence of spatial use differentiation among group members in the giant babax, which may serve as an underlying mechanism for parental care diversification in cooperatively breeding systems. Moreover, our study demonstrates the utility of GPS tracking in monitoring fine‐scale daily activities of tagged individuals, thereby expanding the potential applications of GPS tracking in future behavioral research on small‐bodied passerines.

## Author Contributions

**Ning‐Ning Sun:** data curation (equal), formal analysis (equal), investigation (equal), visualization (equal). **Jian‐Chuan Li:** conceptualization (lead), funding acquisition (lead), project administration (lead), resources (lead). **Rang Li:** data curation (supporting), formal analysis (supporting), investigation (supporting), methodology (equal). **Zhuo‐Feng Li:** data curation (supporting), investigation (equal), methodology (supporting). **Rong‐Yu Xu:** data curation (equal), formal analysis (equal), investigation (equal), visualization (equal). **Miao Cheng:** data curation (supporting), investigation (supporting). **Shu‐Min Wang:** data curation (equal), investigation (equal), validation (equal), writing – review and editing (supporting). **Li‐Qing Fan:** conceptualization (equal), funding acquisition (equal), project administration (equal), supervision (equal). **Bo Du:** conceptualization (lead), formal analysis (lead), supervision (lead), writing – original draft (lead), writing – review and editing (lead).

## Funding

Financial support was provided by the Lasa City Science and Technology Planning Project (LSKJ 202440); Science and Technology Projects of Xizang Autonomous Region, China (XZ202501ZY0018); and the National Natural Science Foundation of China (Grant 32260128).

## Ethics Statement

Research permits: Adult capture and tracking were allowed by the permit licensed by the Forestry and Grassland Bureau of Xizang Autonomous Region, China (2024124).

## Conflicts of Interest

The authors declare no conflicts of interest.

## Supporting information


**Table S1:** Comparisons of the altitudes between dominant females/males and helpers in three annual cycle phases.
**Table S2:** Results of GLMMs examining the variations in different group members' relative contribution to nestling provisioning with nestling age during the breeding phase.
**Table S3:** Results of GLMMs examining variations in the locomotor speeds of tagged individuals with seasonal days and daily times during the breeding phase.
**Table S4:** Results of GLMMs examining variations in the activity levels of tagged individuals with seasonal days and daily times during the breeding phase.
**Table S5:** Results of GLMMs examining the variations in locomotor speed of tagged individuals with seasonal days and daily times during the postfledging phase.
**Table S6:** Results of GLMMs examining the variations in activity levels of tagged individuals with seasonal days and daily times during the postfledging phase.
**Table S7:** Results of fitting GLMMs examining the variations in giant babaxes' locomotor speed with seasonal days and daily times during the wintering phase.
**Table S8:** Results of fitting GLMMs examining the variations in giant babaxes' activity levels with seasonal days and daily times during the wintering phase.

## Data Availability

The data supporting the findings of this study are available in the Zenodo repository, https://doi.org/10.5281/zenodo.18872079.
