# Supplementary material for: Spatial Differentiation Among Family Members of the Cooperatively Breeding Giant Babax Dynamically Adjusts to Temporal Fluctuations in Offspring Demand for Parental Care
Source: Ecol Evol. 2026 Apr 9;16(4):e73484. doi: 10.1002/ece3.73484 (PMC13065895; doi:10.1002/ece3.73484)
Supplement: Supplementary file 1 — Table S1: Comparisons of the altitudes between dominant females/males and helpers in three annual cycle phases. Table S2: Results of GLMMs examining the variations in different group members' relative contribution to nestling provisioning with nestling age during the breeding phase. Table S3: Results of GLMMs examining variations in the locomotor speeds of tagged individuals with seasonal days and daily times during the breeding phase. Table S4: Results of GLMMs examining variations in the activity levels of tagged individuals with seasonal days and daily times during the breeding phase. Table S5: Results of GLMMs examining the variations in locomotor speed of tagged individuals with seasonal days and daily times during the postfledging phase. Table S6: Results of GLMMs examining the variations in activity levels of tagged individuals with seasonal days and daily times during the postfledging phase. Table S7: Results of fitting GLMMs examining the variations in giant babaxes' locomotor speed with seasonal days and daily times during the wintering phase. Table S8: Results of fitting GLMMs examining the variations in giant babaxes' activity levels with seasonal days and daily times during the wintering phase. [file ECE3-16-e73484-s001.docx]

**Supplementary tables**

Table S1. Comparisons of the altitudes between dominant females/males and helpers in three annual cycle phases.

| Model parameters  Fixed effect variables | *β* ± se | *n* | *t* | *P* |
| --- | --- | --- | --- | --- |
| During the breeding phase | | | | |
| Intercept | 3916.962 ± 1.731 | 867 | 2263.451 | < 0.001 |
| ^*^Dominant female | -27.113 ± 3.347 | 104 | -8.101 | < 0.001 |
| ^*^Dominant male | -26.723 ± 2.186 | 478 | -12.223 | < 0.001 |
| Random effect variables | *β* ± SD | *n* | *Z* | *P* |
| Residuals | 853.496 ± 41.064 | 867 | 20.785 | < 0.001 |
| Identity of tagged individuals | 177.787 ± 425.090 | 867 | 0.551 | 0.522 |
| During the offspring post-fledging phase | | | | |
| Intercept | 3909.044 ± 15.693 | 1467 | 249.096 | < 0.001 |
| ^#^Dominant female | -18.571 ± 20.087 | 227 | -0.925 | 0.355 |
| ^#^Dominant male | -34.878 ± 19.547 | 301 | -1.784 | 0.075 |
| Random effect variables | *β* ± SD | *n* | *Z* | *P* |
| Residuals | 2023.664 ± 74.944 | 1467 | 27.002 | < 0.001 |
| Identity of tagged individuals | 360.880 ± 313.778 | 1467 | 1.150 | 0.250 |
| During the wintering phase | | | | |
| Intercept | 3791.556 ± 43.143 | 711 | 87.884 | < 0.001 |
| ^$^Dominant female | 80.704 ± 64.251 | 196 | 1.256 | 0.209 |
| ^$^Dominant male | 41.312 ± 66.517 | 301 | 0.621 | 0.535 |
| Random effect variables | *β* ± SD | *n* | *Z* | *P* |
| Residuals | 3879.631 207.223 | 711 | 18.722 | < 0.001 |
| Identity of tagged individuals | 6727.767 ± 4469.137 | 711 | 1.505 | 0.132 |

Note: ^*^values of helpers were set as the baseline (*n* =285); ^#^values of helpers were set as the baseline (*n* = 939); ^$^values of helpers were set as the baseline (*n* = 275).

Table S2. Results of GLMMs examining the variations in different group members’ relative contribution to nestling provisioning with nestling age during the breeding phase.

| Model parameters  Fixed effect variables | *β* ± SEM | *n* | *t* | *P* |
| --- | --- | --- | --- | --- |
| The relative contribution of dominant males to nestling provisioning | | | | |
| Intercept | 37.995 ± 2.335 | 182 | 16.273 | < 0.001 |
| Nestling age | -1.106 ± 0.263 | 182 | -4.204 | < 0.001 |
| Random effect variables | *β* ± SD | *n* | *Z* | *P* |
| Residuals | 168.974 ± 1.413 | 182 | 9.177 | < 0.001 |
| Nest identity | 13.426 ± 9.984 | 182 | 1.345 | 0.179 |
| The relative contribution of dominant females to nestling provisioning | | | | |
| Intercept | 40.883 ± 2.034 | 182 | 20.097 | < 0.001 |
| Nestling age | -1.379 ± 0.253 | 182 | -5.461 | < 0.001 |
| Random effect variables | *β* ± SD | *n* | *Z* | *P* |
| Residuals | 183.617 ± 19.409 | 182 | 9.460 | < 0.001 |
| Nest identity | 24.604 ± 18.273 | 182 | 1.346 | 0.178 |
| The relative contribution of helpers to nestling provisioning | | | | |
| Intercept | 17.867 ± 2.569 | 182 | 6.956 | < 0.001 |
| Nestling age | 3.067 ± 0.288 | 182 | 10.638 | < 0.001 |
| Random effect variables | *β* ± SD | *n* | *Z* | *P* |
| Residuals | 201.196 ± 22.084 | 182 | 9.156 | < 0.001 |
| Nest identity | 16.808 ± 12.687 | 182 | 1.325 | 0.185 |

Table S3. Results of GLMMs examining variations in the locomotor speeds of tagged individuals with seasonal days and daily times during the breeding phase.

| Model parameters  Fixed effect variables | | *β* ± se | *n* | *t* | *P* |
| --- | --- | --- | --- | --- | --- |
| Intercept | | 1.891 ± 6.075 | 1228 | 0.311 | 0.756 |
| Adult identity | Dominant female | 4.560 ± 8.637 | 104 | 0.528 | 0.598 |
|  | Dominant male | 8.678 ± 8.277 | 887 | 1.048 | 0.295 |
| Adult identity × Seasonal days | Dominant female | -0.023 ± 0.206 | 104 | -0.111 | 0.911 |
|  | Dominant male | -0.104 ± 0.073 | 887 | -1.435 | 0.152 |
|  | Helpers | 0.330 ± 0.148 | 237 | 2.076 | 0.037 |
| Adult identity × Daily times | Dominant female | -0.135 ± 0.208 | 104 | -0.648 | 0.517 |
|  | Dominant male | -0.297 ± 0.070 | 887 | -4.263 | < 0.001 |
|  | Helpers | 0.049 ± 0.145 | 237 | 0.340 | 0.734 |
| Random effect variables | | *β* ± SD | *n* | *Z* | *P* |
| Residuals | | 60.926 ± 2.730 | 1228 | 22.316 | < 0.001 |
| Identity of tagged individuals | | 0.300 ± 6.314 | 1228 | 0.004 | 0.990 |

Note: values of helpers were set as the baseline (*n* = 237).

Table S4. Results of GLMMs examining variations in the activity levels of tagged individuals with seasonal days and daily times during the breeding phase.

| Model parameters  Fixed effect variables | | *β* ± se | *n* | *t* | *P* |
| --- | --- | --- | --- | --- | --- |
| Intercept | | 1.372 ± 0.459 | 11259 | 2.987 | 0.003 |
| Adult identity | Dominant female | -0.069 ± 0.650 | 1330 | -0.107 | 0.915 |
|  | Dominant male | -0.090 ± 0.648 | 7498 | -0.138 | 0.890 |
| Adult identity ×  Seasonal days | Dominant female | 0.125 ± 0.005 | 1330 | 26.383 | < 0.001 |
|  | Dominant male | 0.063 ± 0.002 | 7498 | 31.375 | < 0.001 |
|  | Helpers | 0.085 ± 0.004 | 2431 | 23.655 | < 0.001 |
| Adult identity ×  Daily times | Dominant female | 0.022 ± 0.002 | 1330 | 9.696 | < 0.001 |
|  | Dominant male | 0.049 ± 0.002 | 7498 | 20.927 | < 0.001 |
|  | Helpers | 0.052 ± 0.004 | 2431 | 14.718 | < 0.001 |
| Random effect variables | | *β* ± SD | *n* | *Z* | *P* |
| Residuals | | 0.414 ± 0.007 | 11259 | 61.447 | < 0.001 |
| Identity of tagged individuals | | 0.207 ± 2.070 | 11259 | 0.001 | 0.990 |

Note: values of helpers were set as the baseline (*n* = 2431).

Table S5. Results of GLMMs examining the variations in locomotor speed of tagged individuals with seasonal days and daily times during the post-fledging phase.

| Model parameters  Fixed effect variables | | *β* ± se | *n* | *t* | *P* |
| --- | --- | --- | --- | --- | --- |
| Intercept | | 2.316 ± 2.109 | 266 | 1.10 | 0.273 |
| Adult identity | Dominant female | 0.416 ± 2.586 | 188 | 0.16 | 0.872 |
|  | Dominant male | -2.122 ± 3.945 | 16 | -0.54 | 0.591 |
| Adult identity ×  Seasonal days | Dominant female | 0.001 ± 0.029 | 188 | 0.019 | 0.985 |
|  | Dominant male | 0.156 ± 0.254 | 16 | 0.617 | 0.538 |
|  | Helpers | -0.007 ± 0.034 | 62 | -0.213 | 0.832 |
| Adult identity ×  Daily times | Dominant female | 0.156 ± 0.079 | 188 | 1.982 | 0.049 |
|  | Dominant male | 0.180 ± 0.209 | 16 | 0.861 | 0.390 |
|  | Helpers | 0.077 ± 0.111 | 62 | 0.691 | 0.490 |
| Random effect variables | | *β* ± SD | *n* | *Z* | *P* |
| Residuals | | 17.030 ± 1.502 | 266 | 11.336 | < 0.001 |
| Identity of tagged individuals | | 0.165 ± 6.510 | 266 | 0.010 | 0.990 |

Note: values of helpers were set as the baseline (*n* = 62).

Table S6. Results of GLMMs examining the variations in activity levels of tagged individuals with seasonal days and daily times during the post-fledging phase.

| Model parameters  Fixed effect variables | | *β* ± se | *n* | *t* | *P* |
| --- | --- | --- | --- | --- | --- |
| Intercept | | 2.776 ± 0.321 | 1467 | 8.651 | < 0.001 |
| ^#^Adult identity | Dominant female | 1.794 ± 0.454 | 227 | 3.951 | < 0.001 |
|  | Dominant male | 1.250 ± 0.412 | 301 | 3.036 | 0.002 |
| Adult identity ×  Seasonal days | Dominant female | -0.004 ± 0.001 | 227 | -3.445 | < 0.001 |
|  | Dominant male | 0.003 ± 0.001 | 301 | 3.513 | < 0.001 |
|  | Helpers | 0.004 ± 0.001 | 939 | 8.643 | < 0.001 |
| Adult identity ×  Daily times | Dominant female | -0.022 ± 0.004 | 227 | -5.290 | < 0.001 |
|  | Dominant male | 0.012 ± 0.003 | 301 | 3.681 | < 0.001 |
|  | Helpers | 0.073 ± 0.002 | 939 | 35.632 | < 0.001 |
| Random effect variables | | *β* ± SD | *n* | *Z* | *P* |
| Residuals | | 0.189 ± 0.006 | 1467 | 30.254 | < 0.001 |
| Identity of tagged individuals | | 0.186 ± 0.124 | 1467 | 1.501 | 0.133 |

Note: ^#^values of helpers were set as the baseline (*n* = 939).

Table S7. Results of fitting GLMMs examining the variations in giant babaxes’ locomotor speed with seasonal days and daily times during the wintering phase.

| Model parameters  Fixed effect variables | | *β* ± se | *n* | *t* | *P* |
| --- | --- | --- | --- | --- | --- |
| Intercept | | 9.706 ± 10.062 | 516 | 0.965 | 0.335 |
| Adult identity | Dominant female | 1.431 ± 20.383 | 39 | 0.070 | 0.944 |
|  | Dominant male | -6.937 ± 14.809 | 211 | -0.468 | 0.640 |
| Adult identity ×  Seasonal days | Dominant female | -0.189 ± 0.698 | 39 | -0.270 | 0.787 |
|  | Dominant male | -0.048 ± 0.229 | 211 | -0.212 | 0.833 |
|  | Helpers | -1.192 ± 0.140 | 266 | -8.515 | < 0.001 |
| Adult identity ×  Daily times | Dominant female | -0.396 ± 1.120 | 39 | -0.354 | 0.724 |
|  | Dominant male | 0.072 ± 0.249 | 211 | 0.288 | 0.773 |
|  | Helpers | 0.940 ± 0.241 | 266 | 3.896 | < 0.001 |
| Random effect variables | | *β* ± SD | *n* | *Z* | *P* |
| Residuals | | 0.131 ± 0.009 | 516 | 14.365 | < 0.001 |
| Identity of tagged individuals | | 0.076 ± 0.070 | 516 | 1.084 | 0.278 |

Note: values of helpers were set as the baseline (*n* = 266).

Table S8. Results of fitting GLMMs examining the variations in giant babaxes’ activity levels with seasonal days and daily times during the wintering phase.

| Model parameters  Fixed effect variables | | *β* ± se | *n* | *t* | *P* |
| --- | --- | --- | --- | --- | --- |
| Intercept | | 0.671 ± 0.372 | 588 | 1.806 | 0.071 |
| Adult identity | Dominant female | 0.588 ± 0.587 | 100 | 1.001 | 0.317 |
|  | Dominant male | 0.642 ± 0.503 | 236 | 1.276 | 0.202 |
| Adult identity ×  Seasonal days | Dominant female | 0.158 ± 0.017 | 100 | 9.060 | < 0.001 |
|  | Dominant male | 0.147 ± 0.019 | 236 | 7.678 | < 0.001 |
|  | Helpers | 0.152 ± 0.029 | 252 | 5.267 | < 0.001 |
| Adult identity ×  Daily times | Dominant female | 0.067 ± 0.015 | 100 | 4.334 | < 0.001 |
|  | Dominant male | 0.031 ± 0.009 | 236 | 3.339 | < 0.001 |
|  | Helpers | 0.037 ± 0.009 | 252 | 4.188 | < 0.001 |
| Random effect variables | | *β* ± SD | *n* | *Z* | *P* |
| Residuals | | 0.873 ± 0.051 | 588 | 16.970 | < 0.001 |
| Identity of tagged individuals | | 0.144 ± 0.135 | 588 | 1.072 | 0.284 |

Note: values of helpers were set as the baseline (*n* = 252).
